# Membrane Surface Modification and Functionalization

**DOI:** 10.3390/membranes11110877

**Published:** 2021-11-15

**Authors:** Vivek Vasagar, Mohammad K. Hassan, Majeda Khraisheh

**Affiliations:** 1 PPG Industries, 4325 Rosanna Drive, Allison Park, PA 15101, USA; 2 Department of Chemical Engineering, College of Engineering, Qatar University, Doha 2713, Qatar; m.Khraisheh@qu.edu.qa

With the increase in water scarcity, and as only 2.5% of Earth’s total water is freshwater, numerous researchers have focused on the development of sustainable technology for the generation of freshwater [1]. Membrane technology is one such sustainable technology, which can be used in desalination to produce drinking water, as well as in the separation of gases, the pharmaceutical field, and in wastewater treatment to reclaim water for food crop irrigation. This is due to its advantages, which include a simple operation and scale-up, as well as being an energy-efficient and low carbon footprint process [2,3,4]. Despite its advantages, membranes suffer from fouling and low stability during the separation process. Several studies, including surface modification, have been carried out by researchers to improve the anti-fouling properties and stability of such membranes, thereby increasing the utility of such membranes in separation processes [5,6,7].

Surface modification and functionalization play an important role in several applications by imparting novel properties. The performance properties of a membrane, such as selectivity and flux, are enhanced via surface modification and functionalization, thus increasing its utility. Its performance is increased by minimizing undesired interactions on the surface of the membrane, which is achieved via surface modification and functionalization, which are key factors for anti-fouling properties [6]. Although this is a vital topic in several fields, there are still gaps that needed to be addressed to improve the process of scaling-up, making it viable on a larger scale.

In this Special Issue of *Membranes,* titled “Membrane Surface Modification and Functionalization”, eight research articles are published, including one review paper that emphasizes several aspects—either chemical or physical modifications—that have improved the membrane separation process in a wider field.

In the study by Khraisheh et al. [8], a systematic investigation was carried out to establish a correlation between membrane-fouling characteristics and a fertilizer drawn forward osmosis (FDFO) process. The novelty of this work lies in the utilization of cellulose triacetate (CTA)-based membranes oriented in forward osmosis (FO) mode, while marine aquaculture wastewater and multi-component fertilizer salts were used as feed solutions. Several physical cleaning methodologies, including osmotic backwashing and in situ flushing of the fouled membranes, were studied. The FDFO performance was evaluated by comparing the water flux, percentage recovery, and salt rejection characteristics before and after the physical cleaning process. Water flux showed a decline from 10.32 L/(m^2^ h) to 3.30 L/(m^2^ h) when the feed solution was changed from pure DI water to highly concentrated marine aquaculture wastewater. The water flux through the fouled membrane showed a decrease from 8.6 L/(m^2^ h) to 3.09 L/(m^2^ h) when the marine aquaculture wastewater was used as feed solution, while when using ultrapure DI water, the flux decreased from 13.1 L/(m^2^ h) to 3.42 L/(m^2^ h). Comparing the water flux and water recovery before and after physical cleaning of the foul membranes indicated that these methods were able to reduce the foul layer on the membrane surface. These cleaning methods were able to recover a high percentage of salt, i.e., 75% phosphate and 60% of nitrate salts. Through this work, a fundamental understanding was established by investigating the factors affecting the performance of the membrane in the FDFO process, which can be utilized in membrane synthesis and scale-up processes. Along with this understanding, the recovery effect following physical cleaning methods will enable a high output from FDFO processes. In the work by Asghar et al. [9], a non-thermal atmospheric plasma jet (APPJ) was used to study the inactivation characteristics of microbes. Several factors that affect the efficiency of microbial inactivation, such as discharge current, discharged applied voltage, discharge power, consumed energy injected into the gas, the width of the jet, the axial distance between the APPJ nozzle and the sample, time of exposure, and type of exposure (direct or indirect), were evaluated and found to be a very crucial phenomenon. The disinfection process was also studied with varying concentrations of oxygen in argon gas, and it was found that the wettability of the discharges increased with oxygen concentration, leading to a better inactivation process. Indirect exposure was achieved using a mesh and a magnetic field from the discharges that directly affect the surface. This study demonstrates that the indirect exposure method deals with heat energy transfer to the colonies, which inactivates the media culture in places that are difficult to reach, making this technique very useful in the prevention of virus transmission. 

Liu et al. [10] demonstrated a facile, two-step process to graft chitooligosaccharide (CHO) on the surface of poly(ester-urethane) (SPU) to increase its biocompatibility. In his work, small molecule diisocyanate compounds were reacted with SPU to impart the free NCO groups on the surface. The ~NH_2_ groups from the CHO molecules were utilized to immobilize CHO on the surface. The prepared polymer showed increased hydrophilicity as well as increased hydrolytic degradation, while its mechanical properties were not altered. Furthermore, the SPU-CHO polymer showed increased biocompatibility. Thus, with this study, it was very evident that through the surface grafting of CHO molecules onto an SPU polymer, the authors were able to generate a biodegradable material that can be used for biomedical applications after detailed biological assessments.

Hafiz et al. [11] compared the efficacy between nanofiltration (NF) and reverse osmosis (RO) in purifying municipal wastewater to be used for irrigation. While several studies have assessed the performance of secondary treated wastewater, this study focused on the NF and RO processes to treat tertiary-treated sewage effluent (TSE) to reuse the water for the irrigation of food crops. Since the applied pressure played an important role, water flux, energy consumption and permeate quality were evaluated at varying feed pressures. This comparative study showed that the RO process was suitable for the reclamation of TSE while the NF process was not, due to its low rejection rates for monovalent ions such as Na and Cl, which exceeded the threshold limits recommended by the Food and Agriculture Organization (FAO). In the RO process, the study showed that the highest recovery rate obtained was 16% at an applied pressure of 16 bar, with a specific energy consumption of 0.56 kWh/m^3^. With a slight decrease in applied pressure at 14 bar, there was not much effect on the recovery rate, while the specific energy consumption decreased to 11%. On the other hand, when the applied pressure was further decreased to 12 bar, even though the recovery rate was high, the specific energy consumption was 7% higher, which indicated that the applied pressure needed to obtain an optimal performance was 14 bar. 

Düerkop et al. [12], reviewed several membranes that are targeted for use in vanadium redox flow battery (VRFB) systems. Membranes were categorized and compared based on both the type of polymer used and the type of membrane structure. Several polymers, including Nafion, perfluorosulfonic acid (PFSA) modified membranes, poly(ether ether ketone) (PEEK), poly(sulfone) (PSU), poly(ether sulfone) (PES), poly(phenyl sulfone) (PPSU), poly(vinylidene fluoride) (PVDF), poly(ethylene-tetrafluoroethylene) (ETFE), poly(benzimidazole) (PBI), poly(imide) (PI), perfluorosulfonic acid (PFSA), poly(phenylene ether) (PPE) and poly(tetrafluoroethylene) (PTFE), were compared for their efficiency, cost and cyclic stability. Other physical properties, such as membrane thickness, ion-exchange capacity, water uptake and vanadium-ion diffusion rates, were also compared. This comprehensive review will enable the reader to understand that the performance of VRFB is not exclusively dependent on the membrane used, but also on the type, modification and physical properties of the membranes. 

In the simulation study carried out by Hafiz et al. [13], a hybrid process was analyzed to treat wastewater that is within the allowable standards for food crop irrigation (FAO). This study illustrated a two-step process: the first step is nanofiltration (NF), followed by a second stage, where two different processes, i.e., reverse osmosis (RO) and a hybrid of forward osmosis and reverse osmosis (FO-RO), were studied. The studies indicated that using the process that contained RO in the second step (NF-RO), was not suitable for the further extraction of permeate from the brine generated from the first NF step. This was mainly due to the high salinity of the final permeate at a set recovery rate of 90% with a high total specific energy consumption (Es). With the hybrid approach (NF-FO-RO) in the second step, the Es was 27% lower as compared to the NF-RO process, while the product was within allowable limits set by the FAO at a 90% targeted recovery rate. With these simulation studies, a high throughput can be achieved to purify wastewater for irrigation purposes.

The work by Gryta et al. [14], showed the use of Ar/O_2_ plasma treatment in the surface modification of polypropylene (PP) membranes. This plasma treatment significantly changed the surface to a depth of less than 1 micron, with significant increases in porosity that facilitated mass transport—thus increasing flux within the membrane. This extensive study analyzed the effect of plasma conditions, such as the gas composition, flow rate, plasma power excitation and the time of exposure on membrane flux performance. The properties of the membranes were examined at various time intervals of one, four and five years of storage and compared with the membrane performance immediately after the modification process. The membranes showed good stability and high wetting resistance and were comparable up to a five-year test period. Through this study, it was evident that the optimal membrane properties were obtained under a higher plasma power (205 W), with a gas flow of 90 mL/min. It is worth mentioning that no polymer degradation was observed after plasma treatments.

Several membrane-separation processes, such as forward osmosis, reverse osmosis coupled with nanofiltration and membrane bioreactor processes were employed to separate suspended solids and contaminants in pharmaceuticals and personal care products (PPCPs). In the work by Lin et al. [15], graphene oxide was used to enhance the separation process along with improving the resistance towards H2O2 exposure. In this study, a thin-film composite was made by depositing a polyamide (PA) layer onto commercially available polysulfone support membranes. Graphene was incorporated into the PA layer to exclusively compare its effects with (TFC-GO) and without graphene oxide (TFC). The main objective of modifying the membrane using graphene oxide is to enhance oxidant resistance and to improve separation performance. The permeate flux, monovalent and divalent salt rejection, and the PPCP rejection rates were compared before and after H_2_O_2_ exposure. The membrane was also characterized for its surface morphology, roughness, functional group alterations and hydrophilicity. Scanning electron microscopy images of TFC and TFC-GO membranes showed damage and swelling; however, the permeate flux of TFC-GO was stable, with significantly higher NaCl, MgSO_4_, and PPCP rejection rates, which indicated that the GO in the PA membrane reacts with the oxidants to mitigate membrane surface damage and increase the negative charge on the surface, causing an enhanced electrostatic repulsion of negatively charged PPCPs. This study aids in commercializing GO-based membranes for improved rejection rates, even after exposure to harsh environments.

The articles in this Special Issue cover fundamental research and fill a gap between academic study and commercialization. With knowledge of the factors that influence the performance of such membranes, it could become possible to tailor them for end-use. 

In conclusion, the editors would like to thank the authors and reviewers for their valuable contributions to this Special Issue and the editorial staff of *Membrane Surface Modification and Functionalization* for their help and support during the review process.

## Data Availability

Not applicable.

## References

[1] Yalcinkaya F., Boyraz E., Maryska J., Kucerova K. (2020). A review on membrane technology and chemical surface modification for the oily wastewater treatment. Materials.

[2] Baker R.W. (2012). Membrane Technology and Applications.

[3] Shannon M.A., Bohn P.W., Elimelech M., Georgiadis J.G., Marinas B.J., Mayes A.M. (2008). Science and technology for water purification in the coming decades. Nature.

[4] Bastani D., Esmaeili N., Asadollahi M. (2003). Polymeric mixed matrix membranes containing zeolites as a filler for gas separation applications: A review. J. Ind. Eng. Chem..

[5] Kang M.S., Chun B., Kim S.S. (2001). Surface modification of polypropylene membrane by low-temperature plasma treatment. J. Appl Polym..

[6] Nady N., Franssen M.C., Zuilhof H., Eldin M.S.M., Boom R., Schroen K. (2011). Modification methods for poly(arylsulfone) membranes: A mini-review focusing on surface modification. Desalination.

[7] Zuo G., Wang R. (2013). Novel membrane surface modification to enhance anti-oil fouling property for membrane distillation application. J. Memb. Sci..

[8] Khraisheh M., Gulied M., Almomani F. (2020). Effect of membrane fouling on fertilizer-drawn forward osmosis desalination performance. Membranes.

[9] Asghar A.H., Ahmed O.B., Galaly A.R. (2021). Inactivation of E. Coli using atmospheric pressure plasma jet with dry and wet argon discharges. Membranes.

[10] Liu Y., Liu Z., Gao Y., Gao W., Hou Z., Zhu Y. (2021). Facile method for surface-grafted chitooligosaccharide on medical segmented poly(Ester-urethane) film to improve surface biocompatibility. Membranes.

[11] Hafiz M.A., Hawari A.H., Alfahel R., Hassan M.K., Altaee A. (2021). Comparison of nanofiltration with reverse osmosis in reclaiming tertiary treated municipal wastewater for irrigation purposes. Membranes.

[12] Düerkop D., Widdecke H., Schilde C., Kunz U., Schmiemann A. (2021). Polymer membranes for all-vanadium redox flow batteries: A review. Membranes.

[13] Hafiz M., Alfahel R., Hawari A.H., Hassan M.K., Altaee A. (2021). A hybrid nf-fo-ro process for the supply of irrigation water from treated wastewater: Simulation study. Membranes.

[14] Gryta M., Tomczak W. (2021). Stability of Ar/O_2_ plasma-treated polypropylene membranes applied for membrane distillation. Membranes.

[15] Lin Y.L., Zheng N.Y., Chen Y.S. (2021). Enhancing H_2_O_2_ tolerance and separation performance through the modification of the polyamide layer of a thin-film composite nanofiltration membrane by using graphene. Membranes.

